# Bone Marrow Involvement in a Patient with Alpha Heavychain Disease: Response to Tetracycline Treatment

**DOI:** 10.4084/MJHID.2012.034

**Published:** 2012-05-07

**Authors:** Zahit Bolaman, Irfan Yavasoglu, Gokhan Sargin, Gurhan Kadikoylu, Firuzan Kaçar Doğer

**Affiliations:** 1 Adnan Menderes University, Faculty of Medicine, Division of Hematology, Aydin, Turkey; 2 Adnan Menderes University, Faculty of Medicine, Department of Pathology, Aydin, Turkey

## Abstract

A 28-year-old man from East Mediterranean area admitted with abdominal pain, weight loss and diarrhea. Barium x-ray studies showed segmentation, dilatation of bowel loops, mucosal folds thickening and delayed intestinal transit. Histological examination of biopsy specimens revealed villous atrophy and plasmacytic infiltration limited to mucosa and submucosa. Computed tomography showed multiple lymphadenopathy in the abdomen. Serum protein electropheresis and immunoelectropheresis indicated elevated IgA concentration. Bone marrow aspiration and biopsy revealed presence of lymphoplasmacytic infiltration. Immunohistochemical analysis of the intestine, lymph nodes showed positivity for CD45, CD-79, CD-20. After tetracycline treatment the patient’s symptoms, abdominal lymphadenopathy and bone marrow infiltration disappeared and IgA concentration decreased to normal levels.

## Introduction

Alpha heavy chain disease (α-HCD) was described by Seligmann in 1968.^1^ It is also known as “Mediterranean” lymphoma or immunoproliferative small intestinal disease (IPSID). IPSID is evaluated as a form of mucosa-associated lymphoid tissue (MALT) lymphoma.^2^ In WHO classification of lymphoid and hematopoietic tissue, α-HCD is listed in mature B cell neoplasm as a special form of heavy chain disease.^3^

The classical clinical findings of α-HCD are diarrhea, weight loss and abdominal pain. They may have palpable abdominal masses and exhibiting clubbing.^4^–^6^ The majority of the patients with α-HCD have been reported in Mediterranean, Middle Eastern, Africa and Far East. Common demographic properties of α-HCD are lower socioeconomic status, poor personal hygiene, predominance male sex and a peak age between the first and third decades.^7^,^8^ There is a relation with Campylobacter jejuni and development of α-HCD.^9^

It is characterized by a diffuse and intense plasma cell infiltrate in the lamina propria of the small bowel mucosa along with the synthesis of abnormal alpha-heavy chains without light chains.^10^–^13^ Anemia, vitamin deficiencies and hypogammmaglobulinemia is common. The immunoglobulin A level is generally not increased. Bone marrow involvement of disease such as is very rare.^14^ In the current report, we present a case of α - HCD with bone marrow involvement and complete clinical response to tetracycline treatment in a young male.

## Case Presentation

**Figure 1.**
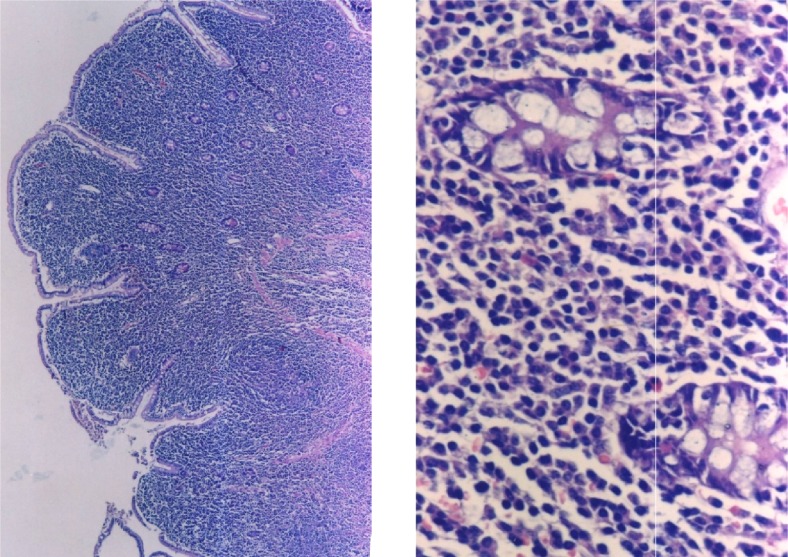
The biopsy sample of the jejunum illustrating complete effacement due to centrocyte like cells and plasma cells in the in mucosa and submucosa (HEx50 and 100)

**Figure 2.**
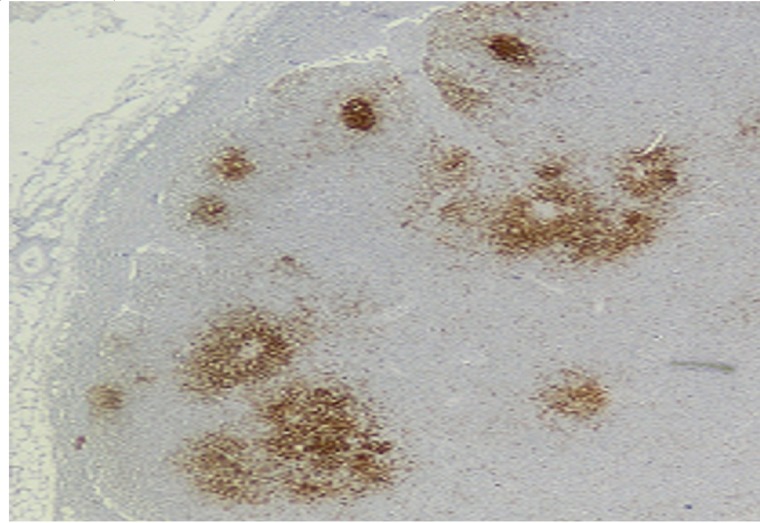
CD 20 staining shows marked positivity of the lymphoid cells noted in the mesenteric lymph node (CD20x100)

**Figure 3.**
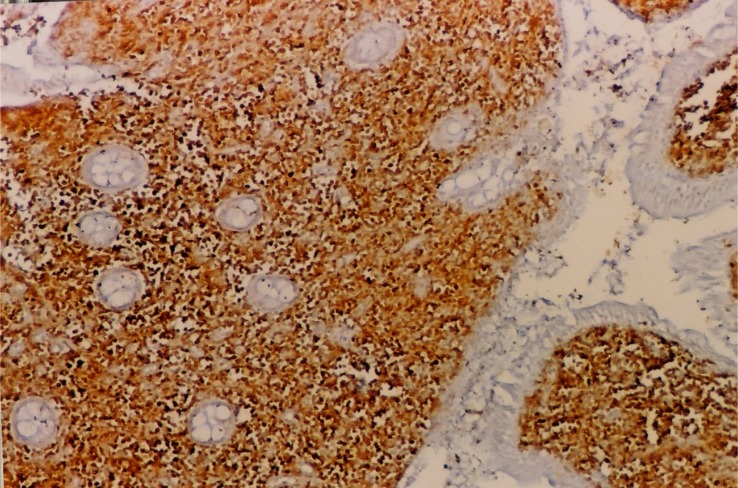
CD 20 staining shows marked positivity of the lymphoid cells noted in the intestinal mucosa (CD20x100)

**Figure 4.**
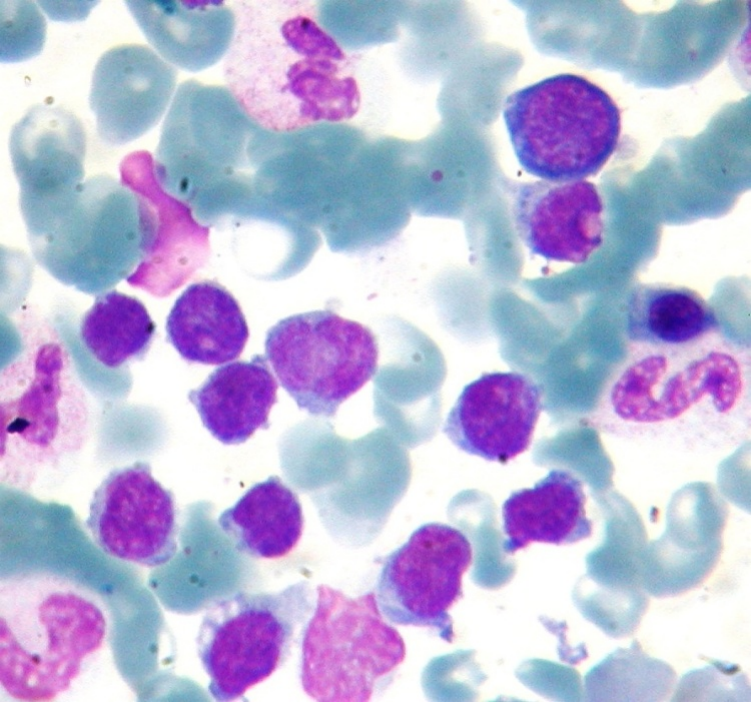
The bone marrow aspirate smear shows markedly increased number of lymphoplasmacytic (MGG stain Oil immersion X1000)

A 28-year-old man from East Mediterranean area admitted to hematology department with complaints of abdominal pain, diarrhea and weight loss of 25 kg in the last six months. On clinical examination, his blood pressure was 110/70 mmHg and pulse rate was 90 beats/min (regular). He was pale with cachectic appearance. He had clubbing of the fingers, edema and evidence of dehydration. There were no palpable lymph nodes or splenomegaly. The patient’s hemoglobin level was 11.6 g/dl, hematocrit level 35.7%, red blood cell count 4.44×10^6^/mm^3^, platelet count 361×10^3^/mm^3^, mean corpuscular volume 80.4 fl mean corpuscular hemoglobin concentration 32.4%, total white blood cells count 22.5x10^3^/mm^3^(lymphocyte count 12.4x10^3^/mm^3^), neutrophil 9.5x10^3^/mm^3^. Lymphocytosis was observed in the peripheral blood smear. Erythrocyte sedimentation rate was 90 mm/h. The multiple plasma biochemical abnormalities included albumin 2.0 g/dl, total protein 5.2 g/dl, low plasma potassium 2.9 mmol/L, serum iron was 42 μg/dl and iron binding capacity 464 μg/dl as well as iron saturation 9%. Serum folic acid level was low while cobalamin and zinc levels were normal. Bacteriological, virological and parasitological studies of stools revealed no evidence for specific agents. Chest x-ray was normal and abdominal x-ray showed air levels in non-dilated large bowel. Barium x-ray studies showed segmentation, dilatation of bowel loops, mucosal folds thickening and delayed intestinal transit. Endoscopic examination showed mild duodenitis. There was no abnormality in colonoscopic examination. Computed tomographic (CT) scans of the abdominal region showed multiple mesenterical lymphadenopathy and thickening of small bowel. Maximal size of lymph nodes were 3 centimetres on CT scan. The samples of duodenal biopsies revealed moderate infiltration by normal appearing plasma cells. Microscopic evaluation of the duodenal biopsy sample revealed no evidence of Helicobacter pylori. The serum IgG, IgA gliadin antibodies and IgA endomysial antibodies were negative. The examination of stool and duodenal aspiration fluid for parasites was normal. The immunological profile was normal except of elevated IgA (**Table 1**). The increased IgA was polyclonal and an abnormal precipitin line of IgA with no response to light chains was found on immunoelectropheresis. In order to determine the extent of disease and to obtain a diagnostic sample laparoscopy was performed. Histological examination of the excised jejunal biopsy showed subtotal villous atrophy and dense lymphoplasmacytic infiltration limited to mucosa and submucosa (**Figure 1**). The extensive plasmacytic infiltaration was established in mesenteric lymph nodes. CD 20 were found positive in the mesenteric lymph node (**Figure 2**). In the immunohistochemical analysis of the intestinal and lymph node biopsy material, LCA, CD-79, CD-20 and IgA were found positive while kappa and lambda heavy chain, IgD and IgM were found negative (**Figure 3**). Bone marrow aspiration and biopsy revealed diffuse lymphoplasmacytic infiltration (**Figure 4**). Malabsorption was confirmed by abnormal D-xylose absorption (urine excretion of D-xylose was 1.20 g/5 h; normal value >4.5 g/5 h). Patient was put on long term treatment with tetracycline regimen (500 mg PO, four time, every day). His symptoms and physical condition improved and diarrhea disappeared at the second week. The patient appeared normal with the exception of clubbing, that was still present at the third month. The lymphocytosis resolved about three months. Follow-up CT of the abdomen showed no abnormality at the sixth month. Serum IgA concentration decreased to normal levels and bone marrow examination performed on the 6^th^ month revealed normal trilineal hematopoiesis without any evidence of lymphoplasmacytic infiltration. Tetracycline treatment continued as one year, as advised in the literature.^4^,^6^ Currently, the patient has been disease-free, in the 8^th^ year after his treatment.

## Discussion

Alpha-heavy chain disease is frequently seen in Mediterranean countries and Middle East.^4^,^10^,^11^ It can involve stomach, colon, liver, peritoneum, periaortic and cervical lymph nodes.^15^ The involvement of bone marrow and increase of immunoglobulin A level in α-HCD is very rare.^14^ The histological findings of α-HCD have been classified into three distinct stages: Stages A includes mature plasmacytic or lymphoplasmacytic infiltration of mucosal lamina propria and mesenteric lymph nodes. Stage B has nodular mucosal infiltrates and infiltrate may extend below the muscularis mucosa. The plasma cells frequently are atypical and some immunoblasts are seen. Stage C consist of lymphomatoid masses and a diffuse “immunoblastic” lymphoma with or without the benign appearing lymphoblastic infiltration seen in stage A.^16^

The presented case had some characteristics of α-HCD disease. The dense infiltration of lymphoplasmacytic in the mucosa and submucosa of jejunum and lymph nodes caused chronic diarrhea and abdominal pain. Immunohistochemical staining of intestine revealed CD45 (LCA), CD-79, CD-20 and IgA positivity (**Figure 1 and 2**). D-xylose absorption test in patient confirmed the absorption disorder. In addition, serum electrophoresis indicated the presence of alpha heavy chain without accompanying light chains. All these data is supportive of the α-HCD.^4^,^17^,^18^ Crohn’s disease and Celiac Sprue was thought in differential diagnosis. Serologic assays and clinical presentation was against the diagnosis of Celiac disease. In Crohn’s disease, microscopic evaluation reveals neutrophilic infiltration and linear ulceration or fissures that were not present in this patient. No granulomatous disease was noted in the histological examination and parasite ova in stool were not seen. Our patient has no antiendomysial antibody and intraepithelial lymphocytosis. Therefore, we excluded Crohn’s disease, Celiac sprue, intestinal tuberculosis and parasitosis. Our patient appeared to belong to stage A lesion because of only lymphoplasmocytic infiltration in mucosa and submucosa with mesenteric nodes without regard to bone marrow involvement. According to our knowledge; there is no consensus about stage of heavy chain disease with bone marrow involvement. In addition, the elevation of immunoglobulin A level an other interesting feature of presented case.

The treatment modality depends on the extent and histological stage of alpha-heavy chain disease. Because of the frequent presence of small intestinal bacterial overgrowth and progression to malabsorbtion, the recommended treatment for stage A has been broad spectrum antibiotics with or without corticosteroids.^14^–^19^ The average complete remission rate treated with antibiotics is reported to be around 30–70% Treatment duration, was six months, minimally.^4^ Some authors suggest a 3 months additional antibiotic treatment, after complete remission.^17^

Our patient responded to tetracycline at second week of treatment and remained symptom free at the third month of tetracycline treatment. Serum IgA level decreased to normal levels and bone marrow infiltration disappeared at sixth month. The lymphocytosis resolved about three months. Due to the clinical presentations and the clinical responses observed in some patients treated with antibiotics, there has been a significant consideration for an unknown organism as the pathogenic factor for the development of alpha chain disease. Using 16S ribosomal sequence as a target, a recent study by Lecuit et al.^9^ demonstrated *Campylobacter jejuni* in the intestinal biopsy from patients with IPSID. The organism was also demonstrated by a probe hybridizing to *Campylobacter jejuni*. Interestingly, one of these patients had a clinical response to antibiotic treatment. We believe the dramatic response to tetracycline therapy noted in our patient fits to the proposed concept of an organism is primarily responsible for this disease. New clinical trials are likely to provide a better understanding of the etiology of alpha chain disease and develop more targeted therapy against *Campylobacter jejuni*.

In the advanced alpha-heavy chain disease, in stage B and C, recommended treatment consists of antibiotics and different chemotherapeutic drug combinations (such as cylophosphamide, doxorubicine, vincristine, bleomycine, prednisolone, nitrogen mustard, procarbasine) and/or total abdominal radiation. Rambaund and Halphen^20^ recommended first-line antibiotics for early stage patients. Patients without marked improvement after a 6-month course of antibiotic or complete remission within 12 months should be given doxorubicin induced chemotherapy. Up to 69% complete remission rate for stage B and C were reported.^11^,^21^ In the literature, the information is very limited in alpha-heavy chain disease with bone marrow involvement. Bone marrow involvement is not effective for the treatment of these patients.^17^,^18^ The bone marrow involvement in alpha-heavy chain disease is very rare. The bone marrow involvement due to alpha-heavy chain disease can be easily differentiated from a number of other disorders presenting with malabsorbtion as there is a typical histopathological view. However, as noted in our patients, it is possible that even in the advanced stage disease antibiotic therapy could be tried as the first line of treatment.

## Conclusion

In summary, we present a rare systemic presentation of the alpha-heavy chain disease with bone marrow involvement. This case illustrates that remission may be possible with the administration of antibiotics such as tetracycline. Therefore, it is critical to initiate a larger study with alpha chain disease in order to determine precise etiology and response rate in this disorder.

## Figures and Tables

**Table 1.** Laboratory data of the patient on admission

***Values***	***Patient***	***Normal***
**Complete Blood Count**		
White blood cell	22.5×10^3^/mm^3^	4–10×103/mm^3^
Neutrophile	9.5 ×10^3^/mm^3^	1.8–7 ×10^3^/mm^3^
Lymphocyte	12.4 ×10^3^/mm^3^	0.9–4.4 ×10^3^/mm^3^
Monocyte	0.5 ×10^3^/mm^3^	0.16–8 ×10^3^/mm^3^
Red blood cell	426×10^3^/mm^3^	450–590 ×10^3^/mm^3^
Hemoglobin	11.6 g/dL	13.5–17.5 g/dL
Platelets	361 ×10^3^/mm^3^	150–350 ×10^3^/mm^3^
Bichemistry		
Blood glucose	95 g/dL	75–115 g/dL
Total protein	5.2 g/dL	5.5–8.0 g/dL
Albumin	2.0 g/dL	3.5–5.5 g/dL
Serum iron	42 μg/dL	50–150 μg/dL
Serum iron binding	464 μg/dL	250–370 μg/dL
Folic acid	2.7 ng/mL	3.1–17.5 ng/mL
Serological tests		
C-reactive protein	6.3 mg/L	0.08–3.1 mg/L
IgA	1870 mg/dL	60–309 mg/dL
IgG	476 mg/dL	614–1295 mg/dL
IgM	20 mg/dL	53–334 mg/dL
Urine		
D-xylose test/5h	1.2 g	>4.5 g
Stool		
Culture	negative	negative
Smear ova	negative	negative
Smear parasite	negative	negative
Bone marrow	lymphoplasmacytic infiltration	no infiltration

## References

[1] Seligmann M (1968). Alpha chain disease: A new immunoglobulin abnormality. Science.

[2] Isaacson PG (1994). Gastrointestinal lymphoma. Hum Pathol.

[3] Campo E, Swerdlow SH, Haris NL (2011). The 2008 WHO classification of lymhoid neoplasms and beyond: evolving concepts and practical applications. Blood.

[4] Al-Saleem T, Al-Mondhiry H (2005). Immunoproliferative small intestinal disease (IPSID): a model for mature B-cell neoplasms. Blood.

[5] Hermans MMH, Klinkhamer P, Stronkhorst A (2001). Malabsorbtion syndrome in a patient of Mediterranean origin; immunoproliferative small intestinal disease. Neth J Med.

[6] Fermand JP, Brouet JC (1999). Heavy chain diseases. Hematol/Oncol Clin North Am.

[7] Khojasteh A, Haghshaness M, Haghighi P (1983). Immunoproliferative small intestinal disease. A “Third-World lesion”. N Engl J Med.

[8] Akbulut H, Soykan I, Yakaryilmaz F (1997). Five- years Results of the Treatment of 23 patient with immunoproliferative small intestinal disease. Cancer.

[9] Lecuit M, Abachin E, Martin A (2004). Immunoproliferative small intestinal disease associated with Campylobacter jejuni. N Engl J Med.

[10] Khojasteh A, Marsh MH (1987). Immunoproliferative small intestinal disease (IPSID) in third world countries. Immunopathology of the small intestine.

[11] Nair S, Mathan M, Ramakrishna BS, Mathan VI (1998). Immunoproliferative small intestinal disease in South India: A clinical and immunomorphological study. J Gastroenter&Hepatol.

[12] Khojasteh A, Haghighi P (1990). Immunoproliferative small intestinal disease: Portrait of a potentially preventable cancer from a the third world. Am J Med.

[13] Isaacson PG, Dogan A, Price SK (1989). Immunoproliferative small intestinal disease: An immunohistochemical study. Am J Surg Pathol.

[14] Nassar VH, Salem PA, Shahid MJ (1978). “Mediterranean abdominal lymphoma” or immunoproliferative small intestinal disease. Part II (pathological aspects). Cancer.

[15] Dispenzeri A, Gertz MA, Greer JP, Foerster J, Lukens JN, Rodgers GM, Paraskevas F, Glader B (2004). Cryoglobulinemia, heavy chain diseases, and monoclonal gammopathy-associated disorders. Wintrobe’s Clinical Hematology.

[16] Galian A, Lecestre MJ, Scotto J (1977). Pathological study of alpha heavy chain, with special emphasis on evaluation. Cancer.

[17] Arista-Nasr J, Gonzalez-R MA, Mantilla-Morales A (1994). Immunoprolifertive small intestinal disease in Mexica. J Clin Gastroenterol.

[18] Rambaud J-C, Brouet J-C, Seligmann M, Ogra PL, Mestecky J, Lamm ME, Strober W, McGhee JR, Bienenstock J (1994). Alpha chain disease and related lymphoproliferative disorders. Handbook of mucosal immunology.

[19] Ben-Ayed F, Halphen M, Najjar T (1989). Treatment of alpha-heavy chain disease. Results of a prospective study in 21 Tunisian patients Tunisian-French Intestinal Lymphoma Study Group. Cancer.

[20] Rambaud J, Halphen M (1989). Immunoproliferativesmall intestinal disease (IPSID): relationships with alpha-chain disease and “Mediterranean” lymphomas. Gastoenterol Int.

[21] O’Keefe SJD, Winter TA, Newton KA (1988). Severe malnutrition associated with α-heavy chain disease: response to tetracycline and intensive nutritional support. Am J Gastroenterol.

